# Applications of 3D-Printed PEEK via Fused Filament Fabrication: A Systematic Review

**DOI:** 10.3390/polym13224046

**Published:** 2021-11-22

**Authors:** Rupak Dua, Zuri Rashad, Joy Spears, Grace Dunn, Micaela Maxwell

**Affiliations:** 1 Department of Chemical Engineering, School of Engineering & Technology, Hampton University, Hampton, VA 23668, USA; zuri.rashad@my.hamptonu.edu (Z.R.); joy.spears@my.hamptonu.edu (J.S.); 2 The Governor’s School for Science and Technology, Hampton, VA 23666, USA; grace.dunn@nhrec.org; 3 Department of Chemistry and Biochemistry, School of Science, Hampton University, Hampton, VA 23668, USA; micaela.maxwell@my.hamptonu.edu

**Keywords:** additive manufacturing, polymer, 3D printing, PEEK, medical, electrical, aerospace, chemical

## Abstract

Polyether ether ketone (PEEK) is an organic polymer that has excellent mechanical, chemical properties and can be additively manufactured (3D-printed) with ease. The use of 3D-printed PEEK has been growing in many fields. This article systematically reviews the current status of 3D-printed PEEK that has been used in various areas, including medical, chemical, aerospace, and electronics. A search of the use of 3D-printed PEEK articles published until September 2021 in various fields was performed using various databases. After reviewing the articles, and those which matched the inclusion criteria set for this systematic review, we found that the printing of PEEK is mainly performed by fused filament fabrication (FFF) or fused deposition modeling (FDM) printers. Based on the results of this systematic review, it was concluded that PEEK is a versatile material, and 3D-printed PEEK is finding applications in numerous industries. However, most of the applications are still in the research phase. Still, given how the research on PEEK is progressing and its additive manufacturing, it will soon be commercialized for many applications in numerous industries.

## 1. Introduction

Polyether ether ketone (PEEK) is an organic polymer with potential applications in various fields and industries, including medical, chemical, aerospace, and electronics industries [1,2,3,4,5,6]. PEEK polymer is obtained by step-growth polymerization of 4,4′-difluorobenzophenone (DFPB) and hydroquinone (HQ) conducted at high temperature in a polar solvent such as diphenyl sulphone, giving them a backbone of ether and ketone molecules [7]. PEEK is a highly durable polymer with an equal balance of a ketone group providing rigidity and an ether group providing flexibility to its structure. PEEK has a wide range of uses in the medical industry due to its excellent biocompatibility, radiolucency, chemical resistance, low density (1.32 g/cm^3^), and mechanical properties resembling human bone [8,9,10]. PEEK competes with many metals and alloys, including titanium and aluminum that serve in manufacturing industries. Being lightweight and having excellent mechanical properties, PEEK has started being used in aerospace industries [3,11]. Additionally, PEEK’s inertness makes it a viable material to construct reactors at the macro and micro levels [12]. PEEK is also a suitable material for customizable parts, given that it is relatively inexpensive and can be transformed into unique structures through additive manufacturing [13,14,15,16].

Additive manufacturing is a technology commonly known as 3D printing that creates a three-dimensional structure through the layer-by-layer deposition of the material. The 3D structure to be constructed using additive manufacturing technology is modeled first using computer-aided design (CAD) software. Then, the CAD generated model file (STL format file) is exported to a slicing software which slices the object layer by layer. This information, along with the printing parameters for 3D printing such as the type of material, temperatures of the extrusion nozzle and chamber, amount of material used in an object, and thickness of each layer in the model, are uploaded to the printer in the form of G code to print [2]. 

PEEK can be printed using two types of 3D printing techniques. Selective laser sintering (SLS) and Fused Filament Fabrication (FDM) or fused filament fabrication (FFF). SLS is a process in which thermal energy provided by a laser or an electron beam selectively fuses regions of a powder bed together in a layer-by-layer manner to create the solid structure [17], while in FDM printing technology, the filament is loaded the printing system, typically through a feeder motor. Then, it is heated to a semi-liquid state and is extruded through a nozzle where it is printed on a bed in a layer-by-layer manner until the whole object is formed. The resolution of SLS printers can be reach 50–100 μm; however, they are very expensive compared to FDM printers. Therefore, FDM printers are widely used even though they operate at a higher resolution of 100-150 μm [17].

The printed model may sometimes undergo a post-production treatment process, such as polishing or grit-blasting to change surface texture [2]. PEEK filaments can be made and reinforced with many materials such as carbon fiber (CF) [18] and printed using additive manufacturing with ease [15,19]. The result of a 3D-printed PEEK object is a chemically stable, dense, and rigid material that can be produced in a matter of hours, and the simplicity of the additive manufacturing process has dramatically increased the application of PEEK for customizable parts in numerous industries. 

The practicality of 3D-printed PEEK has been a focal point of study for many researchers in numerous industries, particularly in the past decade [1,20,21,22]. This article reviews the current status of 3D-printed PEEK that has been used in various fields due to its excellent properties. We will also emphasize the current challenges in printing the PEEK and its inspiration for the future. Most of the literature included in this review paper was published after 2010, and there are dozens of discoveries every day due to the ubiquity and reasonable cost of 3D printing. 

## 2. Materials and Methods

### 2.1. Inclusion Criteria

The review paper includes the following article types: original research studies and review articles in which structures made of 3D-printed PEEK and their composites have been used for applications in various fields, including the medical, chemical, aerospace, and electronics industries. All publications included in this analysis were written in English. 

### 2.2. Exclusion Criteria

Studies involving PEEK or their composites not manufactured using 3D printing were excluded in discussing applications of 3D-printed PEEK in this review. Studies that involved 3D-printed PEEK but did not elaborate on the application to a specific industry were also excluded from the consideration.

### 2.3. Search Strategy

**Figure 1:**
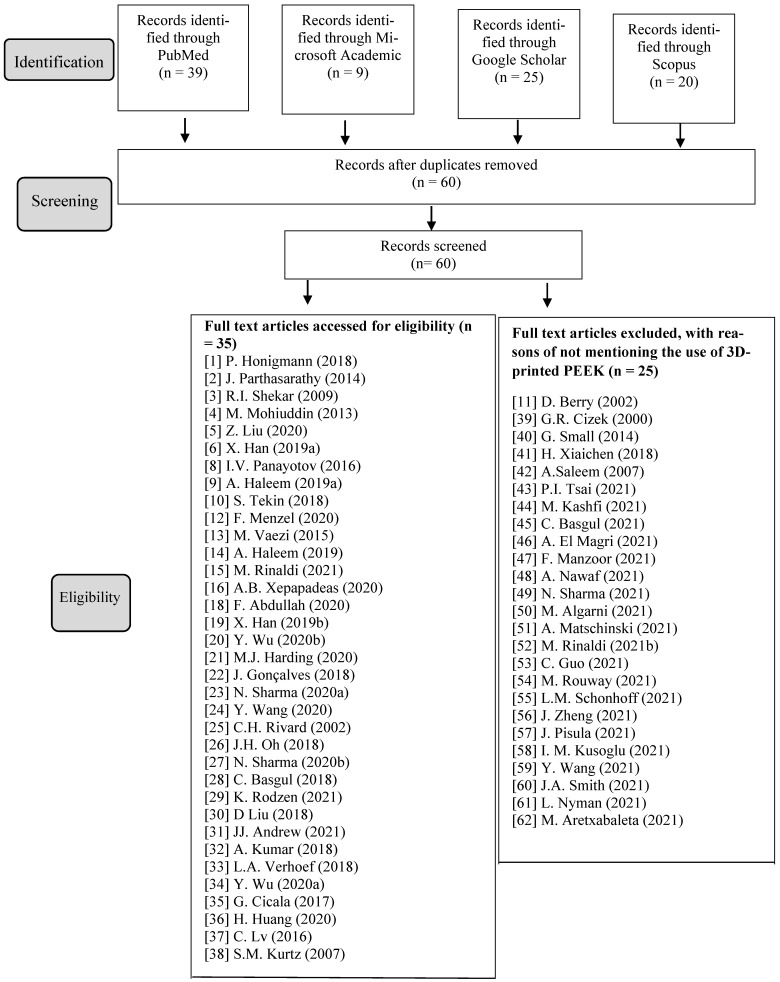
Flow diagram of the different stages of the systematic review [1,2,3,4,5,6,7,8,9,10,11,12,13,14,15,16,17,18,19,20,21,22,23,24,25,26,27,28,29,30,31,32,33,34,35,36,37,38,39,40,41,42,43,44,45,46,47,48,49,50,51,52,53,54,55,56,57,58,59,60,61,62].

Three examiners conducted the search in an independent manner. The following databases were searched: PubMed, Microsoft Academic, Google Scholar and Scopus. The keywords used were as follows: “PEEK” OR “polyetheretherketone” AND “Additive manufacturing”; “3D printed PEEK applications”; “3D printed PEEK applications” AND “medical” OR “chemical” OR “aerospace” OR “electrical”. Appropriate changes to the keywords were made to follow the syntax rules of each database. There was no restriction as regards the year of publication. The search was concluded in September 2021. A flowchart for the systematic review is presented in Figure 1.

## 3. Classification of Use of Additively Manufactured/3D-Printed PEEK in Different Fields

### 3.1. Use of 3D-Printed PEEK in the Medical Field

PEEK is well known for its high-temperature, semi-crystalline, chemically inert, lightweight, high thermal stability, and mechanical properties [1,2,6]. This, along with intrinsic properties of PEEK, such as it having no artifacts in medical imaging and its cortical bone-like young’s modulus, making it an excellent alternative for metallic biomaterials in orthopedics, spine and craniomaxillofacial reconstructive surgeries, spinal fusion, and bone screws [19,23,24]. Additionally, Rivard et al. found that PEEK is a safe material for use in surgery, since there was no trace of infection properties or issues with cell regrowth around the material [25].

**Figure 2:**
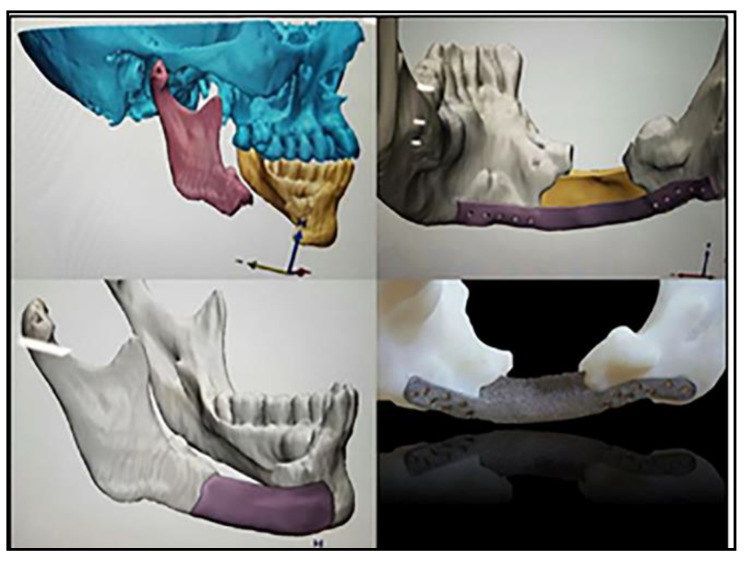
Pictures showing pre-operative diagnosis, virtual surgery and creation of patient-specific implants using CAD/CAM software [26]. Reproduced with permission from Ji-hyeon Oh, Maxillofacial Plastic and Reconstructive Surgery published by Springer Nature, 2018.

PEEK can be additively manufactured or “3D-printed” using the 3D printers that are small and compact enough to be put inside hospitals and other medical facilities [23]. The 3D-printed PEEK allows the development of customized patient-specific implants (PSIs) to fit precisely into the defect space, which cannot be accomplished using traditional manufacturing practices. There are numerous design methods used to recreate a model of the implants with ease using computer-aided design (CAD) or computer-aided manufacturing (CAM) technologies. Some of the design methods using CAM/CAD are printing a pre-operative model with pre-operative data or printing a cutting guide or template after virtual surgery or printing an implant model after virtual surgery with reconstructed data using a mirror image or manufacturing PSIs of both small and large sizes by directly obtaining PSI data after reconstruction using a mirror image [26] (Figure 2).

These 3D-printed PEEK implants have been shown to provide faster implant production, including shorter pre-operative planning and surgery times, postoperative complication reduction, and shorter patient hospitalizations [27]. In comparison, metallic implants can lead to prosthetic loosening, streak artifacts in the CT scan images from the metal’s increased ability to absorb radiation, and limited possibility of post-examining the patient with metallic implants using conventional MRI techniques [1,39]. Basgul et al. tested 3D-printed intervertebral lumbar cages made from PEEK for mechanical strength. They found that the 3D-printed cages formed using fused filament fabrication (FFF) can provide sufficient strength for lumbar cage applications in spinal fusion surgeries. Their research also demonstrated the correlation between printing speed and porosity, with higher rates leading to higher porosities [28].

A research study was conducted on the design and 3D printing of a palatal plate to correct abnormalities in newborns. This research group found that manual adjustments such as hand-cutting or smoothing were necessary to optimize a functional ridge along the vestibular fold and remove support structures. The additively manufactured (3D-printed) plate fits better than the traditional plate obtained from subtractive manufacturing. They recommended the feasibility and implementation of additive manufacturing into the clinical routine to treat neonates and infants with craniofacial disorders [16]. 

**Figure 3:**
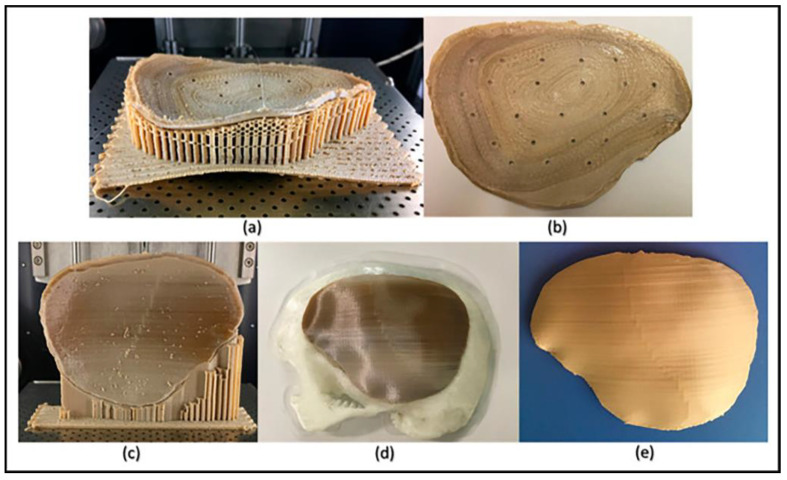
Illustrations of the FFF PEEK 3D printing issues in the cranial implants regarding different orientations. (**a**) Horizontally printed cranial implant showing the raft detachment/warping effect (in situ); (**b**) horizontally printed cranial implant displaying a rough internal surface; (**c**) vertical printed cranial implant exhibiting different levels of crystallinity (in situ); (**d**) 3D-printed skull biomodel with the vertically printed implant after support structure removal; and (**e**) annealed vertically printed cranial implant displaying no discolorations [23]. Reproduced under the Creative Commons Attribution License permission from an open access *Journal of Clinical Medicine* published in 2020.

To evaluate the dimensional accuracy of the FFF or fused deposition modeling (FDM) 3D printer in fabricating complex anatomically shaped structures using PEEK for PSIs, one research group printed the 3D model of a patient’s skull obtained via a CT scan. The fabricated model was then digitized using an optical-based scanning system, and a 3D comparison analysis was conducted. They found that the dimensional accuracy of the printed implant had a mean difference ± SD of 0.03 ± 0.60 mm, which was within the clinical acceptance range for craniofacial reconstructions and were printed in a short period (<24 h) [27]. In a different study, Sharma et al. studied how other characteristics of 3D-printed implants, including layer thickness, infill rate, the number of shells, the orientation of model on the bedplate, the temperature, and infill patterns may affect the quality of the final product. Their result showed that the dimensional deviations were mainly affected by the orientation of the printed model, infill pattern, and the print was least affected by the infill rate. The optimum parameters to print PEEK were a layer thickness of 150 μm, infill rate of 80%, number of shells of 2, and a rectilinear infill pattern. Attention must be paid to temperature control during the printing process to ensure implants of uniform crystallinity [23] (Figure 3). 

Han et al. investigated the surface roughness, wettability, cell adhesion, metabolic activity, and proliferation on 3D-printed PEEK implants. In their study, PEEK disk samples were fabricated with a FFF 3D printer and were modified with polishing and grit blasting. The three groups (untreated, polished, and grit-blasted) were evaluated for their surface modification. Their results revealed that FFF 3D printing exhibited high surface roughness and optimal printing structures, leading to higher cell metabolic activity and cell proliferation than polished and grit-blasted samples [6]. 

PEEK composites can also be 3D-printed that can further enhance the properties of pure PEEK. Rozeń et al. studied the feasibility of 3D printing a PEEK/Hydroxyapatite (HA) composite using FFF printing methods. Using a co-rotating twin-screw extruder, the research group successfully manufactured PEEK/HA composites up to 30 wt.% HA. However, the mechanical properties of the printed PEEK/HA composites became slightly inferior when moving beyond 20–30 wt.% HA. They also found that the optimal temperature for operating the printer of 370 °C and the chamber temperature of 200 °C resulted in PEEK/HA samples with a tensile modulus of 4.1 GPa [29].

Han et al. studied carbon-fiber-reinforced (CFR) 3D-printed PEEK composites and compared them to pure PEEK. Using an FDM printer, this group printed the PEEK and CFR-PEEK samples with dimensions set according to ISO standards to evaluate the mechanical properties (tensile, bending, and compressive tests). They printed the samples with the extrusion temperature set to 420 °C and the printing speed to 40 mm/s. Their results revealed that the PEEK samples with reinforced carbon fiber had significantly better tensile and bending strengths than PEEK. Still, they did not find any statistical difference in compressive strength [19]. 

Three-dimensional-printed PEEK prosthetics can be especially helpful to patients seeking treatment for scapular benign fibrous histiocytoma, or bone tumors. Liu et al. used X-rays, and CT scans to observe a damaged left shoulder joint with osteolytic bone destruction. Then, a left shoulder scapular prosthetic was 3D-printed made from PEEK to fit the cancer patient. After three months after the insertion of the patient-specific implant and removal of the tumor, no trace of the autogenous scapula was found, and the patient had a normal amount of joint rotation with the new prosthetic. This trial concluded that 3D-printed PEEK was a satisfactory option for total replacement of bone tissue in cancer patients [30].

According to Haleem et al., one of the unique fields where PEEK has a promising future is dentistry [14]. Additive manufacturing of PEEK implants helps confront challenges prevalent in orthodontics, such as the need for customizable implants fitted to a specific patient and the high cost of the implant material. Additive manufacturing creates a mold based on a three-dimensional scan of the person’s mouth. Additionally, the layer-by-layer extrusion process of fused deposition modeling wastes far less material than the machining process of creating implants [14]. By reducing the cost of production and constructing more accurately fitted prosthetics for patients, 3D-printed PEEK would positively impact the field of orthodontics as a whole.

Three-dimensional-printed PEEK medical devices and surgical tools have also made an impact on medical research. These printed medical devices not only have the benefits of PEEK but are also shown to be remarkably similar to regularly manufactured devices. However, there is still a long way to transition from researching 3D-printed PEEK to integrating it into treatment within clinics [24]. Nevertheless, PEEK has shown promising results in the medical field, especially in implant and medical device manufacturing.

### 3.2. Use of 3D-Printed PEEK in the Aerospace Field

PEEK is also used in the aerospace field and is a favorable material in this industry due to its excellent properties, including lightweight, mechanical properties, strong thermal stability, resistance to ultra-violet (UV) radiation, and chemicals [3,18]. PEEK is used as a thermoplastic resin and heat-resistant structural component in aerospace applications [3].

PEEK components manufactured using additive manufacturing are finding a special place in the aerospace industry. In this industry, it is necessary to use lightweight materials with intense physical and thermal properties. Rinaldi et al. utilized the fused deposition modeling (FDM) method, a form of additive manufacturing, to print a nanosatellite for space applications using PEEK polymer. The nanosats are a class of satellites that are miniature or small in size with low mass. Due to the lightweight nature PEEK, and additive manufacturing technology, the investigators provided a proof of concept for the nanosat structure composed of 3D-printed PEEK. The investigators in their study also tested the outgassing parameters of 3D-printed PEEK that included RML (recovered mass loss), TML (total mass loss), WVR (water vapor regained), and CVCM (collected volatile condensable material). Their results showed that the aerospace industry’s requirements were fully met, establishing that the 3D printing PEEK does not negatively impact outgassing properties. Thus, 3D-printed PEEK is suitable for space technology [15].

**Figure 4:**
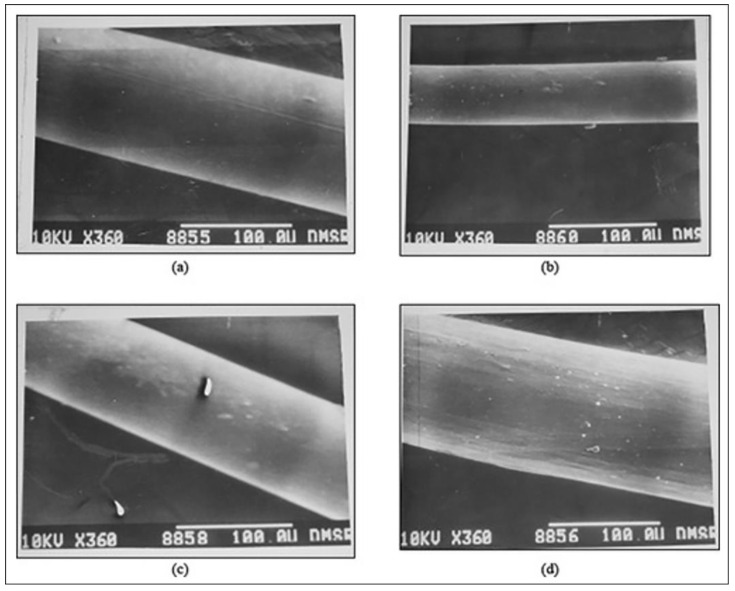
SEM photographs of extruded and drawn PEEK yarns at different magnifications. The photographs showed that the filament is cylindrical in shape with uniform diameter, has a smooth surface, and is transparent with no irregular striations on its surface [3]. Reproduced under the Creative Commons Attribution License from an open-access *Journal of Clinical Medicine* published in 2020.

In an attempt to design a high modulus PEEK fiber for aerospace applications, one of the other studies related to the extrusion and drawing of PEEK fibers found that the crystalline structure in 3D-printed PEEK can be altered with changes in draw temperatures. To manufacture the PEEK filament that is being used in 3D printers, the extruded PEEK filament is passed through a winding unit before it reaches the drawing plate, where the temperature is manipulated. The temperature of this drawing plate is the draw temperature. As the draw temperature decreases, crystal lattice decrease was observed by the investigators. An extensive range of fibers can be printed by utilizing a diverse range of draw temperatures. Shekar et al., in their study, found that a drawing temperature of 200 °C provided an optimal crystallinity level to PEEK and concluded that the 3D-printed PEEK had a similar tensile strength to other fibers utilized in the aerospace industry (Figure 4) [3].

Moreover, it was also found that Nylon and the PEEK fiber have similar sonic velocity properties, demonstrating that PEEK is a suitable material for high temperature and strength-bearing applications [3].

A very recent study proposed to manufacture a heat shield to protect spacecraft when entering Earth or another planetary atmosphere using a 3D-printed carbon fiber/PEEK (CF/PEEK) material instead of the current labor-intensive method for manufacturing heat shields. Three-dimensional printing will help to reduce cost and increase efficiency in the production of the heat shield [18]. In their study, the 3D-printed CF/PEEK heat shield test samples were subjected to intense ultraviolet (UV) radiations, thermal cycles, and high-temperature environments. They were evaluated for their mechanical properties after the exposure. They found that the 3D-printed CF/PEEK material held firm under these extreme radiation environments and showed no significant change to its modulus or tensile strength. Overall, Abdullah et al. showed that the 3D CF/PEEK exhibited excellent recession resistance while maintaining their strong mechanical properties even under extreme environmental conditions, making this material a potential candidate suitable for use as a heat shield material for spacecraft applications [18].

Another research group used the same FDM method for 3D printing to construct nuclear shielding materials. Their study found that 3D printing PEEK and Tungsten composites allows for cheaper, more efficient, and lighter shields that provide more protection against low-energy gamma rays than higher energy [20]. Similarly, PEEK composites are used as neutron absorbers. In another study Wu et al. built a Boron PEEK composite utilizing the FDM method of additive manufacturing. That study found that the PEEK possessed strong and fast neutron shielding properties. Similar to Wu’s work on gamma-ray shielding, PEEK in the neutron shielding application is also a low-cost, lightweight, and efficient material [34].

Temperature properties are crucial in aerospace applications, especially for instruments that will be involved in the extreme temperatures of the re-entry to Earth’s atmosphere. It was found that the 3D-printed PEEK devices have some thermal issues and may produce hotspots [15]. However, when exposed to thermal cycles, 3D-printed PEEK reinforced with carbon fibers showed decreased tensile strength, without any significant change in the young’s modulus [18].

### 3.3. Use of 3D-Printed PEEK in the Electrical Field

PEEK has recently begun emerging in the electrical field as well. PEEK has a lot of potential in the electrical area, as PEEK can become conductive when reinforced with different materials [36,42].

Saleem et al., in their investigation, studied the electrical resistivity and thermal conductivity of PEEK and carbon fiber composites. They found that PEEK becomes electrically conductive when carbon fiber is added to it [42]. In their study, Huang et al. reinforced this idea, where they ground the recycled carbon fiber (rCF) and PEEK in the ratio 1:10 into a powder form that was extruded into a composite filament of diameter 1.75 mm. Furthermore, they used FDM printers to develop the fabrication parts from the composite filament and studied the electrical properties of the formed parts. They compared the electrical properties of pure PEEK with their newly synthesized rCF–PEEK composite. On measuring the electrical conductivity with an electric analyzer, they found that the electrical conductivity of the rCF incorporated PEEK showed a 96.69% improvement over pure PEEK [36].

**Figure 5:**
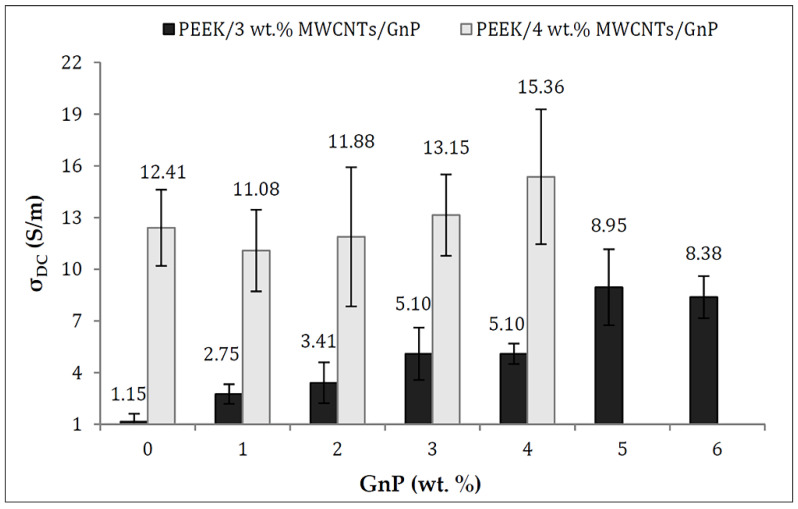
Direct Current (DC) volume electrical conductivity of PEEK/MWCNT/GnP nanocomposites as a function of GnP (1 to 6 wt%) content (at CNT contents of 3 and 4 wt %) [22]. Reproduced under the Creative Commons Attribution License from the *Journal of Applied Polymer Science* published in 2009.

Another research group investigated the electrical properties of PEEK incorporating carbon nanotubes (CNT) and graphite nanoplates developed using FDM processing. They found that the 3D-printed PEEK fused with three to four percent CNT and one to six percent graphene nanoplates had a high electrical conductivity in the range of 1.5–15.36 S/m and maintained this conductivity at high-temperature environments of up to 200 °C (Figure 5). Additionally, the inclusion of up to five percent graphene nanoplates did not affect the characteristic smoothness of the PEEK filament [22].

Andrew et al. investigated the piezoresistive properties of CF-PEEK by 3D printing different structures of CF-PEEK (hexagonal, chiral, and re-entrant), etching the surfaces with concentrated sulfuric acid, and placing the samples on an MTS electronic universal testing machine (UTM) equipped with a load cell of 300 kN” in order to quasi-compress the samples at 5mm/min. The electrical resistance of each sample was measured via a digital multimeter. The researchers discovered that applying stress would increase the piezoresistivity of the CF-PEEK samples between gauge factors of one and three for in-plane compression and between one and five for out-of-plane compression. CF-PEEK was determined to be a material with a high level of piezoresistivity that could potentially serve as an electronic sensor for load-bearing devices [31].

### 3.4. Use of 3D-Printed PEEK in the Chemical Field

Being inert and resistant to most of the chemicals, PEEK has also been utilized in chemical industries. PEEK is widely used in fabricating parts for liquid chromatography applications, including tubing, fittings, pump unions, column hardware, etc. [37]. Being customizable and easy to additive manufacture, 3D-printed PEEK is also finding potential uses in reaction chemistry and fabrication of reactors. Current chemical reactors are made with metal; however, the high cost of production makes them very expensive. Reactors made from additive manufacturing of PEEK are considered a more accessible option due to their excellent mechanical strength, high melting point, excellent chemical resistance, and the ability to create complicated internal channels that would not be possible using traditional manufacturing processes [21]. Additionally, researchers are finding a way to improve upon PEEK by using chemical processes to modify the surface of PEEK to increase its efficacy [37].

Harding et al., in their research, investigated the performance of 3D-printed PEEK reactor components developed using the FFF process for use in liquid-liquid extraction and flow chemistry. After designing the flow reactor parts using CAD software, parts were printed using an FFF printer, and their internal structure was evaluated using X-ray Micro-computed tomography (µCT). The printed parts were also analyzed for mixing performance, pressure resistance, and flow chemistry suitability. This research found that the FFF method can be used to print highly customizable flow reactors parts that can withstand the pressure of 30 bar even with superheated solvents without any leakage. Furthermore, they also demonstrated that the µCT technique used in their study for quality assurance could be adopted to comply with the production environment [21].

Another research group also developed the 3D-printed PEEK Milli and microfluidic reactors mixers and parts and then evaluated their mixing performance for use for flow reactions at elevated temperatures. They found that the mixer with parallel crossed barriers in the channel displayed the best mixing performance. In their study, they also demonstrated the possibility of printing microreactors with channel dimensions below 500 μm. Their design revealed that 3D-printed reactor designs are cheaper, more customizable, and instantly available [12].

## 4. Discussion

We identified a total of 93 articles in this systemic review article. Following the removal of duplicates, 60 were screened, out of which 35 matched the inclusion criteria. After reviewing the articles, we found that the printing of PEEK is mainly conducted by fused filament fabrication (FFF) or fused deposition modeling (FDM) printers owing to its ease and cost. We also summarized the growing use of 3D-printed PEEK in medical, aerospace, electrical, and chemical fields (Table 1).

The most prevalent application for 3D-printed PEEK based on the literature search is within the medical field. PEEK has numerous advantages within the medical field, one of which is its biocompatibility, which allows it to be implemented surgically into the human body without causing any toxic effects [16]. The other advantage is its ability to be additively manufactured without changing its biocompatibility, which provides a higher degree of accuracy within patient-specific implants than a metallic implant would. It also allows patients to undergo routine exams like CT scans and MRIs without producing image artifacts [35]. Finally, its mechanical strength and its elastic modulus, relatively close to that of human bone, prevent the problem of stress shielding that leads to the failure of metallic implants [38]. Furthermore, PEEK can be 3D-printed with reinforced material such as carbon fiber to produce a material with more excellent tensile and bending strength that can be used to create future medical devices [19]. One of the limitations of non-printed PEEK polymer is its bio inertness, so it does not allow cells to attach to it, leading to poor bone osseointegration. However, 3D-printed PEEK yields roughed surfaces suitable for cells to adhere to [19]. One of the drawbacks of PEEK is that it absorbs moisture, so if a reusable medical device is made using PEEK, it will be subjected to repeated sterilization processes. Hence, a change in the mechanical properties of PEEK needs to be accounted for it to be used at a commercial level [32].

This review article also identified several studies that demonstrated the use of 3D-printed PEEK for the aerospace industry. One of the most significant challenges in the aerospace field is producing a vehicle that can exert enough power to exit Earth’s atmosphere. Therefore, the aerospace industry tries to minimize the weight of aircraft and load-bearing parts on board. Using PEEK in place of heavy metal fixtures helps increase the flight capability while keeping the same strength and temperature control of materials. Reducing the projectile’s weight will reduce the amount of fuel required for travel too [33]. Hence, replacing many unique parts in an aircraft with specialized 3D-printed PEEK parts creates a lighter spacecraft. Furthermore, PEEK’s thermal stability and resistance to UV radiation and chemicals [15] make it an excellent candidate to be the base for nanosatellites, heat shields, and nuclear shielding, especially when reinforced with carbon fiber. [15,20,34]. In addition, recycling carbon fiber as a reinforcement material with PEEK during 3D printing also benefits the environment by producing less carbon fiber waste [36].

The electronics field frequently utilizes conductive materials and PEEK’s versatile properties in the electrical field, as certain reinforcement materials allow PEEK to become highly conductive. In this review article, we found four studies that used 3D-printed PEEK for electrical applications. PEEK was reinforced with carbon fiber and carbon nanotubes, which dramatically increased the conductivity over pure PEEK [21,33]. Along with conducting electrons, PEEK also has the ability to absorb neutrons [34], which introduces the polymer to fields that study high-frequency light waves such as gamma rays.

A non-reactive material, such as PEEK, combined with the ease of printing customizable fittings, permits PEEK to be formed into reactors for various processes, such as liquid chromatography, liquid–liquid extraction and flow chemistry [12,21,37]. As FDM 3D printers can operate at the scale of micrometers, PEEK can even be used to build microreactors with high degrees of precision using 3D printing.

The mechanical properties of the additive manufactured PEEK models are relatively lower than the conventional processing methods of developing PEEK, such as injection molding. The mean tensile strength of PEEK manufactured using injection molding ranges from 75 to 220 MPa depending on the blend of PEEK [63] and is a function of mold temperature [64]. In comparison, experimental results from additive manufactured PEEK were found to have a mean tensile strength of 73 MPa [63]. The lower mechanical properties of the additive manufactured PEEK models compared to their injection-molded counterparts are attributed to the poor inter-layer bonding, lower packing density, and the presence of voids [65]. Furthermore, the mechanical properties of additive manufactured PEEK can also be easily altered based on the orientation of printing the model without any additional cost. Rahman et al., in their study, conducted tensile testing for PEEK at three raster orientations, 0°, 90° and alternating between 0° and 90°. They found that the 0° samples demonstrated stronger tensile properties and were weakest for the 90° samples. This is because the mechanical loading direction and the printing orientation are the same for 0° orientation, while this direction was normal for 90° [63].

PEEK is also being used in the automobile industry because of its excellent mechanical strength [40,41]. PEEK also can absorb energy, which increases safety when operating automobiles because the impact of any potential collision would be better absorbed by the car instead of being transferred to the driver, which reduces casualties in the long run [31]. However, we could not find any study of the use of 3D-printed PEEK in the automobile industry. In the future, we expect the use of 3D-printed PEEK will increase in the automobile industry and other fields in order to fabricate materials and designs with intricate designs that will not be possible using traditional manufacturing processes. Furthermore, we expect that there will be many new reinforcement materials coming under research that will be used to further enhance the properties of 3D-printed PEEK.

Though PEEK is customizable and can be manufactured using additive manufacturing, care must be taken while printing PEEK. Depending on the manufacturer of the PEEK filament, PEEK can melt at different temperatures and solidify once extruded at various speeds. The speed of the extruder nozzle of the 3D printer needs to be adjusted to allow the structure to form as intended and settle before an additional layer is applied [23]. When the 3D printer operates too quickly, the layers of the printed polymer can merge, therefore damaging the quality of the final product [15]. Wang et al. found that increasing the printing speed and layer thickness negatively affected all mechanical properties of fiber-reinforced PEEK composites. In comparison, a lower printing speed can aid printing stability and promote extrusion and adhesion of high viscosity PEEK composites. Different printing factors other than print speed, such as layer thickness, nozzle temperature, bed and chamber temperature, impact material failures. The tensile strength and flexural strength of CF/PEEK and GF/PEEK will increase with nozzle and platform temperature increase, likely because the higher nozzle temperature makes for better melting fluidity and formability of printed materials. Additionally, higher platform temperatures produce more energy which improves the infiltration and diffusion among filaments and interlayers. Further from this review, we also found higher tensile and flexural strength of FDM printed 5wt% CF/PEEK and 5wt% GF/PEEK when compared with FDM-3D-printed pure PEEK, due to the strong pinning effect of the fibers; however, the impact property of fiber-reinforced PEEK composites reduced as more fibers were introduced [64].

In this literature review, we did not analyze the overall environmental impact of PEEK compared to other materials. Although recycled carbon fiber can be integrated in PEEK in certain applications [36], further research would be needed to measure the sustainability of PEEK in the future.

As with any systematic review article, there may be some bias in selecting the studies, so this review article may have excluded a few articles that may have matched the selection criteria. We did not provide any quantitative data in this article such as meta-analysis of the articles that are more standardized and less subjective.

## 5. Conclusions

Based on the results of this systematic review, it is concluded that PEEK is a versatile material, and 3D-printed PEEK is finding applications in the medical, aerospace, electrical, and chemical fields. However, most of the applications are still in the research phase. Still, from how the research on PEEK is progressing and its additive manufacturing, it will soon be commercialized for many applications in numerous industries.

## Figures and Tables

**Table 1:** Summarizing the application and use of 3D-printed PEEK in different fields.

Field of Use of 3D-Printed PEEK	Properties of PEEK	Material	Applications	References
Medical	Young’s modulus similar to bone Biocompatible Customizable Excellent mechanical properties	PEEK PEEK/HA Carbon-fiber reinforced PEEK (CF/PEEK)	Craniofacial reconstruction Intervertebral lumbar cages Palatal plates Surgical tools and devices Dentistry	[6,14,16,19,23,24,27,28,29,30]
Aerospace	Light weight Thermal stability Chemical and UV resistance	PEEK CF/PEEK Boron reinforced PEEK	Nanosat Heat Shield Nuclear Shield	[3,15,18,20,34]
Electrical	Conductive	PEEK reinforced with carbon nanotubes and graphite nanoplates Carbon-fiber reinforced PEEK	Use of conductive properties of PEEK at high temperature environments	[22,31,36]
Chemical	Chemical Resistance Inert	PEEK	Liquid Extraction Fabrication of reactors Reaction and flow chemistry	[12,21,37]

## Data Availability

The data presented in this study are available on request from the corresponding author.
